# Antimicrobial activity of essential oils extracted from *Litsea cubeba*

**DOI:** 10.48130/FR-2022-0002

**Published:** 2022-02-25

**Authors:** Xue Wang, Ming Gao, Liwen Wu, Yunxiao Zhao, Yangdong Wang, Yicun Chen

**Affiliations:** 1 State Key Laboratory of Tree Genetics and Breeding, Chinese Academy of Forestry, Beijing 100091, People's Republic of China; 2 Research Institute of Subtropical Forestry, Chinese Academy of Forestry, Hangzhou 311400, Zhejiang Province, People's Republic of China

**Keywords:** Essential oil crop, Families, Chemical component, Antimicrobial activity, Diversity

## Abstract

*Litsea cubeba* (Lour.) Pers. (Lauraceae), also known as May Chang tree or Chinese pepper, is frequently utilized for its essential oil, which is widely used in flavors, perfumes, and antimicrobials. Despite its myriad of uses, the stability and diversity of the various chemical components of *L. cubeba* oil have not been sufficiently investigated. Here, we utilized 31 families planted in a test forest. The stability of the essential oil content in each family was assessed over a four-year period. The chemical profiles of the essential oils from the 31 families were established. A total of 103 components were identified, with approximately 30 components found per family. Additionally, the antifungal and antibacterial activities were investigated, with significant variations found among families. The most abundant component was citral, which has previously been shown to possess antifungal activities. In addition, inhibition rates, EC50, and MIC values were measured, and the F7, G3, G4, and F9 families were found to manifest significantly stronger antifungal activity, with inhibition rates above 91% at a concentration of 250 µL/L. The F7, G3, G4, and L24 families possessed strong antibacterial activity on gram-negative bacteria at a concentration of 50 µL/mL. In summary, we assessed the chemical profiles of *L. cubeba* essential oil for different families and found that there were significant differences in essential oil components and antibacterial activities among families. Our results suggest that *L. cubeba* families can be further selected to improve their industrial applications and increase the quality of essential oils extracted from them.

## INTRODUCTION

In recent years, consumers have become increasingly concerned about synthetic chemical additives, fertilizers, and pesticides. In order to identify safe and sustainable alternatives, a wide range of natural products have been examined. The recent desire for alternative, naturally derived antimicrobials has led to a renewed scientiﬁc interest in plant essential oils, which are considered a safer alternative to synthetic additives. Essential oils can be used in place of synthetic additives^[1]^, insecticides^[2]^, mycotoxin inhibitors^[3]^, and plant pathogen inhibitors^[4]^. *Litsea cubeba* (Lour.) Pers., also referred to as May Chang tree or Chinese pepper, is a type of deciduous tree belonging to the family *Lauraceae,* which is widely distributed in Southeastern Asia, Southern China, Japan, and Taiwan^[1,5]^. *L. cubeba* can be used for flavoring, antimicrobials, and ornamental purposes. In addition to its antioxidant properties, *L. cubeba* has been reported to have uses in the treatment of human diseases, including gastrointestinal discomfort, respiratory diseases, and bacterial infections^[6,7]^. In addition, it can produce volatile essential oils that are extractable via water distillation. A yellow essential oil extracted from its fruit, called *L. cubeba* oil, is insoluble in water and has an odor similar to a mixture of lemon, pepper, and ginger. In addition, the *L. cubeba* oil is used as a raw material in perfumes, as well as the manufacture of citral, vitamins A, E and K, ionone, methylionone and other essential oil mixtures^[8]^.

*L. cubeba* oil also has fungicidal activities against *Sclerotinia sclerotiorum*, *Thanatephorus cucumeris*, *Pseudocer-cospora musae* and *Colletotrichum gloeosporioides*^[8]^. In addition, the essential oils from different *L. cubeba* organs have been shown to prevent the growth of bacteria, likely due to components contained in their essential oils^[6]^. *L. cubeba* oil has been shown to possess significant antimicrobial activities against *Vibrio parahaemolyticus*, *Listeria monocytogenes*, *Lactobacillus plantarum* and *Hansenula anomala*^[1]^. *L. cubeba* oil has strong antifungal activity due to its ability to inhibit mycelial growth and alter ultrastructures in *Aspergillus ﬂavu*, it is considered a safe plant-based preservative^[9]^. In product processing, *β*-cyclodextrin (*β*-CD) is used as a shell material for the manufacture of *L. cubeba* oil microcapsules^[10]^. Due to its myriad of uses, it is critical to gain a better understanding of the underlying components which result in *L. cubeca* antibacterial activities.

In order to identify *L. cubeba* plants with strong antifungal and antibacterial activities, 31 families of *L. cubeba* trees in an eight-year-old testing planation were selected and tested for their efficacy against *Fusarium oxysporum f. sp. fordii* 1 (*Fof*-1), while 12 other families were also tested against *Escherichia coli* and *Listeria monocytogenes*. Essential oil compositions in all families were tested using a Gas Chromatography-Mass Spectrometer (GC-MS). Statistical analyses were then employed to identify families with good antifungal and antibacterial properties.

## RESULTS

### The stability of the essential oil content in *L. cubeba* families

Correlation analysis of the essential oil content in families was conducted, which revealed a significant positive relationship for each family that were sampled from 2014 to 2017 (Table 1). This result indicated that the components of each family were relatively stable across time, which is consistent with a previous report indicating essential oil content heritability ranged from 0.700 to 0.928^[11]^. The stability of essential oil profiles therefore indicates that breeding for desirable compositions is possible.

**Table 1:** Correlation between yield per plant and oil content across different years.

Related age	Yield per plant (Coefficient)	Oil content (Coefficient)
2014−2015	0.79**	0.69**
2014−2016	0.72**	0.77**
2014−2017	0.82**	0.47**
2015−2016	0.71**	0.77**
2015−2017	0.75**	0.56**
2016−2017	0.77*	0.49**
* indicates a significant correlation (significance level of 5 %), ** indicates a highly significant correlation (significance level of 1 %).		

### Chemical profiles of essential oils from *L. cubeba* families

**Figure 1:**
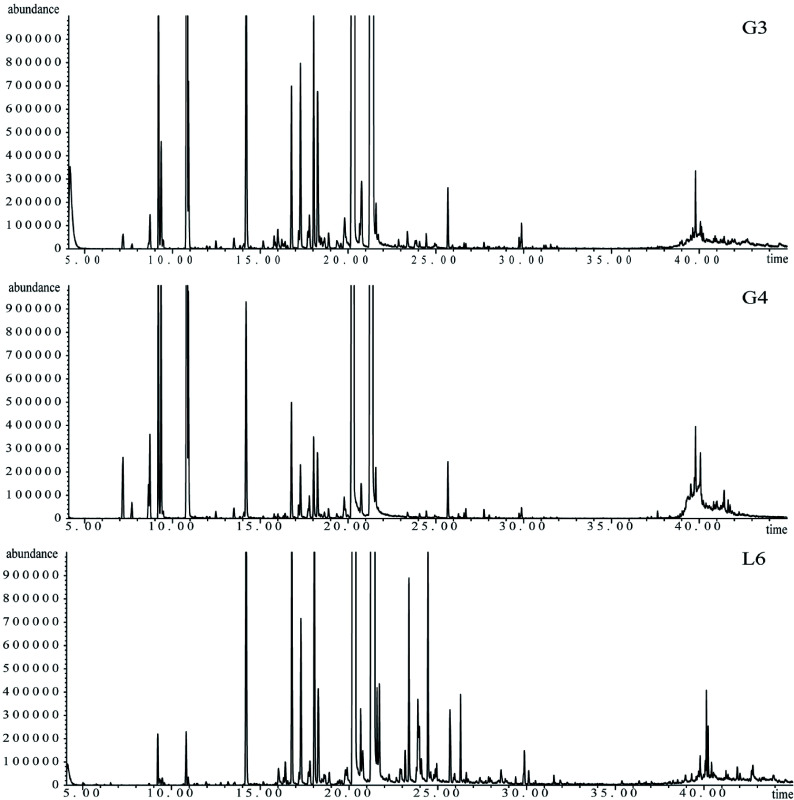
GC–MS ion flow diagrams of essential oils from *L. cubeba* families G3, G4, and L6. The x-coordinate represents retention time in min, while the y-coordinate represents ion abundance.

A total of 103 chemical components from the oils of 31 *L. cubeba* families were examined by GC-MS. Most families contained 30 to 40 components and the percentage of total ingredients was above 99.8%. The total ion flow diagrams of G3, G4, and L6 *L. cubeba* oils are shown in Fig. 1. All the ion flow diagrams showed similar quasi-molecular ions and the base peaks appeared within 20 to 27 minutes. Differences were seen among the number and strength of peaks, especially from 10 to 20 minutes. A total of 63 components belonged to monoterpenes, 40 of which belonged to oxygenated monoterpenes (Table 2). There were 10 sesquiterpenes and only one diterpene in the essential oils from the 31 *L. cubeba* families. Of all the 103 chemical compounds, only seven were common in all 31 families, which indicated a large amount of diversity in the essential oils. Consistent with previous studies, monoterpenes were the dominant components and were mainly represented by neral and geranial^[12]^. All contents of essential oil components from the 31 *L. cubeba* families can be found in Supplemental Table S1 and Table S2. There were 11 abundant ingredients in *L. cubeba* oils, including geranial, neral, D-Limonene, caryophyllene, citronellal, linalool, α-Terpineol, nerol, geraniol, 5-hepten-2-one, and eucalyptol, although these were not detected in every family. The most abundant constituent was geranial (36.74%−48.95%), followed by neral (31.81%−43.55%). The content of D-limonene ranged between 0.03% and 9.64%. The content of geranial was highest in the L6 family (50.00%) and the content of neral was highest in the L24 family (44.00%). In addition, the content of geranial was lowest in F8 (36.74%), while neral was lowest in F11 (31.81%). In the families of L28, L24, L9, L21, L27, and L19, the contents of both geranial and neral were found to be more than 40%.

**Table 2:** Classification and statistics of chemical constituents in oil extracted from 31 *L. cubeba* families.

	Monoterpene hydrocarbons	Oxygenated monoterpenes	Sesquiterpenes	Diterpenes	Others
Numbers	24	40	10	1	29
Total content	0.03%−11.92%	79.78%−97.45%	0.21%−6.23%	0.05%−0.43%	0.34%−12.45%

### Antifungal activity assessments for the 31 *L. cubeba* families

**Figure 2:**
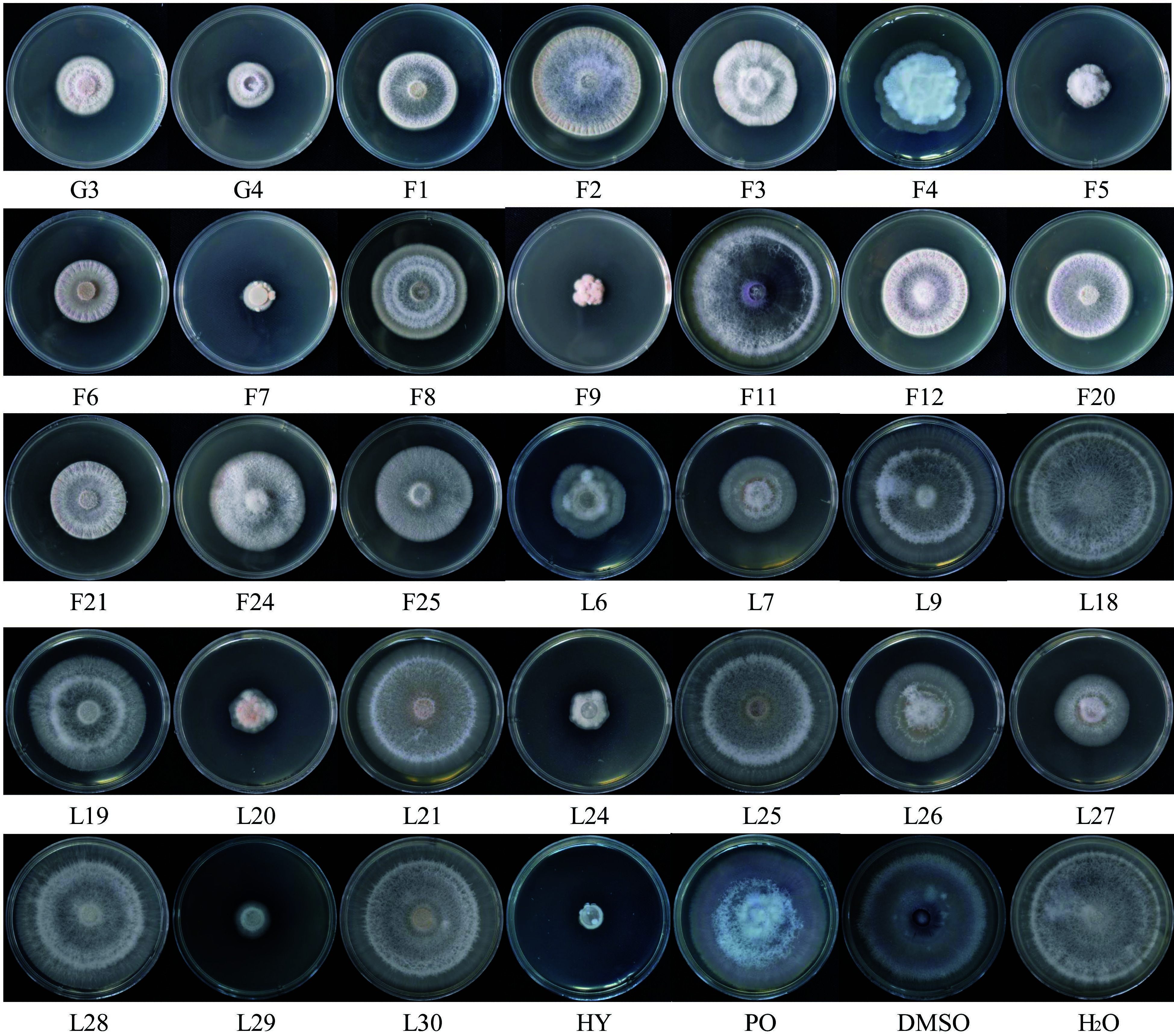
The morphology of *Fof*-1 cultured with *L. cubeba* essential oils from families G3−L30. The pathogen *Fof*-1 was cultured at PDA medium with the *L. cubeba* essential oils from 24 families at the concentration 250 µL/L in 7 days, the picture was recorded using a Digital Single Lens Reflex camera (Nikon D610). G3−L30 represented the families of *L. cubeba*. The 'HY' and 'PO' were represented for two inorganic antifungal and antibacterial agents (hygromycin and polyoxin, respectively). The 'H2O' expressed that water was used instead of essential oils as a control test. Petri dishes with diameter 90 mm were used.

To assess the effect of DMSO on *Fof*-1, the fungal pathogen was cultured in PDA medium with DMSO (DMSO-PDA medium) for six days. The growth state of *Fof*-1in DMSO-PDA medium was the same as that in PDA medium, with the mycelium filling the petri dish after six days of growth. *Fof*-1 pathogen was then added to PDA medium containing the essential oils from the 31 *L. cubeba* families, hygromycin and polyoxin, with five gradient concentrations. All colony morphologies were recorded using a Digital Single Lens Reflex camera (Nikon D610) after seven days of growth. The effects of seven days of 250 µL/L of *L. cubeba* oils from each family are shown in Fig. 2. In general, the 31 families exhibited differences in their antifungal effects. The families G3, G4, F1, F5, F6, F7, F9, F21, L6, L7, L20, L24, and L29 exhibited stronger antifungal properties, while F11, L9, L18, L21, L25, L28, and L30 showed weak antifungal effects. In comparison to the two antibiotics, hygromycin had a better antifungal effect than polyoxin. In addition, the antifungal activities of *L. cubeba* essential oils were better than polyoxin, while the essential oil of F7 showed even stronger inhibitory effects than hygromycin.

The inhibition rate, toxicity equation, goodness of fit (R²), minimal inhibitory concentration (MIC), and the median effect concentration (EC50) for *L. cubeba* essential oils from the 31 families and the exogenic antifungal and antibacterial agent (hygromycin and polyoxin, respectively) are shown in Table 3. Of all the tested samples, the F7, G3, F9, F4, and L6 families had the lowest MIC concentration, with values below 500 µL/L. The MIC values for L25, F11, and L26 families, as well as polyoxin, were more than 1,000 µL/L. For EC50 assessments, the values in G4, L19, F5, G3, F3, F25, F9, L28, and L29 families, as well as hygromycin, were all below 100 µL/L, while the values of the F8, L25, L7, and F11 families, as well as polyoxin, were all over 200 µL/L. The MIC and EC50 values in the G3 family were 265.5 µL/L and 63.3 µL/L, respectively. For the F9 family, the MIC and EC50 values were 321.1 µL/L and 73.4 µL/L. In addition, the MIC and EC50 values were 1,177.4 µL/L and 213.2 µL/L in the L25 family, and they were 1,284 µL/L and 345 µL/L in the F11 family. The MIC and EC50 of polyoxin were 17,581 µL/L and 1,642 µL/L. Overall, the G3 and F9 families had the best antifungal activity, while the L25 and F11 families, as well as polyoxin, had the lowest antifungal effects.

**Table 3:** Assessment of antifungal activities of essential oils produced by different *L. cubeba* families.

Families	Inhibition rate %				Toxicity equation	R^2^	MIC µL/L	EC50 µL/L
	62.5 µL/L	125 µL/L	250 µL/L	500 µL/L				
HY	60.76%	74.06%	81.95%	98.20%	y = 0.401x + 0.2868	0.9616	600.6	34.0
PO	−10.74%	−2.37%	8.82%	22.25%	y = 0.4856x − 0.5758	0.9920	17,581.6	1,642.1
F1	11.62%	28.98%	63.71%	96.57%	y = 0.962x − 0.6978	0.9816	581.9	175.8
F11	−6.38%	10.06%	22.47%	64.21%	y = 0.8761x − 0.8473	0.9423	1,284.0	345.0
F12	13.33%	29.10%	62.50%	97.41%	y = 0.9489x − 0.6779	0.9750	586.5	174.3
F2	10.01%	33.98%	60.39%	97.84%	y = 0.963x − 0.6957	0.9884	576.6	174.4
F20	10.78%	21.23%	66.18%	94.75%	y = 0.9861x − 0.7477	0.9542	592.0	184.2
F21	9.30%	25.81%	65.33%	97.85%	y = 1.0138x − 0.7688	0.9771	555.5	178.5
F24	26.42%	49.56%	70.58%	99.05%	y = 0.7937x − 0.376	0.9959	541.6	127.0
F25	49.04%	59.73%	72.91%	92.36%	y = 0.4755x + 0.092	0.9810	812.0	72.1
F3	49.57%	57.75%	65.10%	89.64%	y = 0.4561x + 0.1105	0.9808	891.7	71.4
F4	7.16%	34.70%	83.53%	100.00%	y = 1.2686x − 0.9736	0.9747	359.5	145.1
F5	53.85%	63.20%	75.36%	95.19%	y = 0.4523x + 0.1548	0.9700	739.0	58.0
F6	9.39%	27.78%	71.21%	97.64%	y = 1.0237x − 0.762	0.9784	526.3	170.9
F7	5.60%	62.46%	98.37%	100.00%	y = 1.5408x − 1.1353	0.9833	243.1	115.2
F8	7.80%	25.73%	49.65%	91.58%	y = 0.9143x − 0.7036	0.9616	729.9	207.2
F9	44.78%	67.54%	91.76%	100.00%	y = 0.7803x − 0.1756	0.9997	321.1	73.4
G3	48.01%	76.79%	96.37%	100.00%	y = 0.8031x − 0.1437	0.9881	265.5	63.3
G4	84.36%	86.54%	92.03%	98.23%	y = 0.1565x + 0.7077	0.9621	737.4	0.5
L18	11.15%	38.14%	61.36%	90.77%	y = 0.8743x − 0.5879	0.9954	654.9	175.5
L19	66.64%	71.21%	80.47%	94.55%	y = 0.2992x + 0.4075	0.9719	955.6	20.4
L20	37.88%	58.00%	67.58%	95.56%	y = 0.6066x − 0.1092	0.9668	673.8	101.0
L21	15.43%	38.87%	65.01%	85.97%	y = 0.7091x − 0.3835	0.9862	893.4	176.2
L24	25.71%	44.99%	59.72%	91.41%	y = 0.7037x − 0.3233	0.9732	759.4	147.9
L25	17.66%	31.70%	49.24%	79.43%	y = 0.6738x − 0.3954	0.9674	1,177.4	213.2
L26	30.22%	39.81%	55.00%	71.96%	y = 0.4664x − 0.0894	0.9857	2,166.5	183.5
L27	23.69%	42.85%	68.82%	90.26%	y = 0.7497x − 0.3712	0.9970	674.5	145.2
L28	46.00%	58.72%	75.90%	88.69%	y = 0.4825x + 0.0714	0.9963	840.5	77.3
L29	43.06%	53.56%	63.83%	98.84%	y = 0.6369x − 0.111	0.9760	555.1	91.1
L30	38.83%	50.15%	71.76%	85.69%	y = 0.5387x − 0.056	0.9866	912.6	107.7
L6	34.74%	56.89%	78.87%	100.00%	y = 0.7234x − 0.2261	0.9999	495.4	100.9
L7	9.78%	28.95%	49.78%	87.46%	y = 0.7777x − 0.5401	0.9757	955.7	217.5
L9	34.92%	53.04%	69.14%	97.84%	y = 0.6805x − 0.2115	0.9819	603.0	111.1
Annotations: 'HY' and 'PO' represent hygromycin and polyoxin, respectively. Based on the toxicity equation, the MIC value was used as the 'x' value when the 'y' value was set as one. Similarly, EC50 value was used as the 'x' value when the 'y' value was set as 0.5. Lower values of MIC and EC50 indicate stronger antifungal effect.								

### The antifungal curve and significance analysis of *L. cubeba* oils

**Figure 3:**
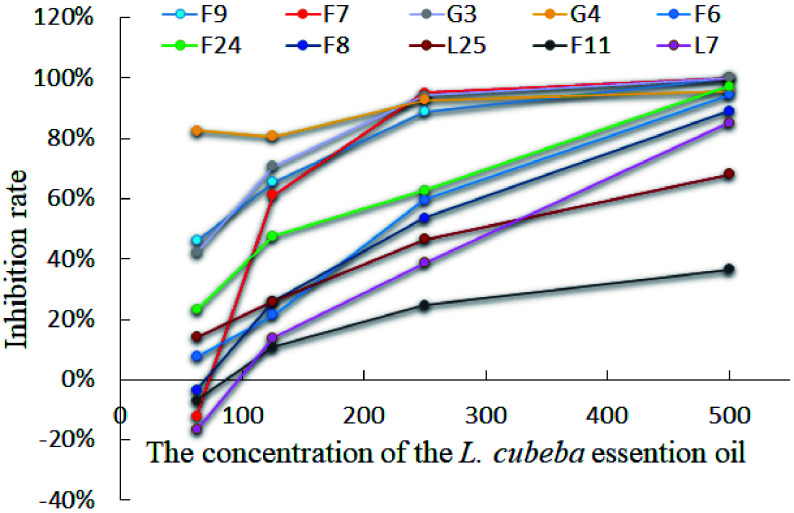
The inhibition rate of *L. cubeba* essential oils from different families with gradient concentrations against *Fof*-1 for 6 days. Lines with different colors represent the different families, including F9, F7, G3, G4, F6, F24, F8, L25, F11, and L7.

In order to further explore the antifungal effect of *L. cubeba* essential oils, the inhibition rates at different times and concentrations were used to plot antifungal curves. Based on the antifungal activity assessments of *L. cubeba* essential oils, F9, F7, G3, G4, F6, F24, F8, L25, F11, and L7 families were utilized for additional analysis. These families are denoted with different colors in the graph displayed in Supplemental Fig. S1. This analysis showed that the inhibition rate of essential oils increased with increasing concentration (Supplemental Fig. S1a−c). Most samples reach a peak effect at a concentration of 250 µL/L of the essential oil, followed by a leveling off. In addition, the patterns of inhibition rate were similar at different times (Supplemental Fig. S1d), which indicated that the cultivation time had little influence on the antifungal activities of *L. cubeba* oils. The antifungal curve of *L. cubeba* oils did differ with concentration (Fig. 3). The inhibition rate in the G4, G3, F9, and F7 families was stable at higher concentrations (> 80%, 250 µL/L), which indicated strong antifungal activity. However, the inhibition rate differed in families when the concentration of essential oil was less than 250 µL/L. In particular, the inhibition rate of F7 increased dramatically from less than 20% to 100% as concentration increased. The inhibition rate of G4 remained above 80% but never reached 100%.

We next employed DPS to further analyze the significant differences among the essential oils from the 31 different families. The significance values of the inhibition rates were calculated at a concentration of 250 µL/L (Table 4). The F7, G3, G4, and F9 families, as well as hygromycin, were shown to have significantly stronger effects than all other treatments, with the exception of essential oils from F4. In addition, the antifungal properties of oils extracted from the F4, L19, L6, L28, F5, F25, L30, F6, F24, L9, L27, L20, F20, F21, F3, L21, and F1 families were significantly better than that of L7, F8, L25, and F11 families, as well as polyoxin. As shown in Table 4, the essential oil extracted from the F7 family had the best antifungal activity, with an inhibition rate of 98.37%, while F11 had the worst inhibition rate (22.47%).

**Table 4:** The significance tests of *L. cubeba* essential oils at a concentration of 250 µL/L.

Essential oils number	Inhibition rate %				5 % significance level	1% significance level
	96 h	120 h	144 h	Average		
F7	100.00%	100.00%	95.10%	98.37% ± 2.83%	a	A
HY	100.00%	98.27%	96.32%	98.2% ± 1.84%	a	A
G3	98.32%	96.54%	94.25%	96.37% ± 2.04%	a	AB
G4	91.41%	92.06%	92.64%	92.03% ± 0.62%	ab	ABC
F9	95.93%	90.60%	88.75%	91.76% ± 3.73%	ab	ABC
F4	87.17%	84.77%	78.67%	83.53% ± 4.38%	bc	BCD
L19	83.67%	82.11%	75.62%	80.47% ± 4.27%	cd	CDE
L6	78.30%	79.28%	79.02%	78.87% ± 0.5%	cde	CDEF
L28	79.14%	76.47%	72.09%	75.9% ± 3.56%	cdef	DEFG
F5	77.31%	76.56%	72.21%	75.36% ± 2.76%	cdefg	DEFGH
F25	77.98%	75.36%	65.39%	72.91% ± 6.64%	cdefgh	DEFGHI
L30	76.36%	71.74%	67.17%	71.76% ± 4.59%	defghi	DEFGHI
F6	82.90%	71.05%	59.70%	71.21% ± 11.6%	defghi	DEFGHI
F24	77.00%	72.08%	62.65%	70.58% ± 7.29%	defghij	DEFGHI
L9	72.97%	69.93%	64.54%	69.14% ± 4.27%	efghijk	EFGHIJ
L27	72.18%	72.55%	61.72%	68.82% ± 6.15%	efghijk	EFGHIJ
L20	67.54%	67.62%	67.59%	67.58% ± 0.04%	fghijk	EFGHIJ
F20	77.05%	66.33%	55.15%	66.18% ± 10.95%	fghijk	FGHIJ
F21	74.68%	65.94%	55.37%	65.33% ± 9.67%	fghijkl	FGHIJ
F3	67.07%	66.17%	62.07%	65.1% ± 2.66%	ghijkl	FGHIJ
L21	69.00%	65.53%	60.49%	65.01% ± 4.28%	ghijkl	FGHIJ
F1	75.99%	62.92%	52.23%	63.71% ± 11.9%	hijkl	GHIJK
F12	76.73%	61.25%	49.52%	62.5% ± 13.65%	hijkl	GHIJKL
L18	70.48%	63.05%	50.56%	61.36% ± 10.07%	ijkl	HIJKL
L29	60.03%	63.83%	59.34%	61.07% ± 2.42%	ijkl	IJKL
F2	69.46%	61.51%	50.20%	60.39% ± 9.68%	jklm	IJKL
L24	63.32%	60.78%	55.04%	59.72% ± 4.24%	klmn	IJKL
L26	60.22%	55.95%	48.82%	55% ± 5.76%	lmn	JKL
L7	61.38%	49.18%	38.79%	49.78% ± 11.31%	mn	KL
F8	43.13%	52.20%	53.60%	49.65% ± 5.68%	n	KL
L25	51.96%	49.31%	46.47%	49.24% ± 2.75%	n	L
F11	20.25%	22.55%	24.61%	22.47% ± 2.18%	o	M
PO	15.79%	24.24%	26.73%	22.25% ± 5.73%	o	M
LSD tests were used for analysis of variance (ANOVA). Lowercase letters indicate 5% significance, while uppercase letters indicate 1% significance. Identical letters for treatments indicate that they were not significantly different when compared. Two letters, such as 'cd', indicate that there are significant differences among all treatments that contain neither 'c' or 'd'. The symbol '±' indicates the average value of the standard deviation among comparisons.						

### Correlation of oil components with antifungal activity

**Figure 4:**
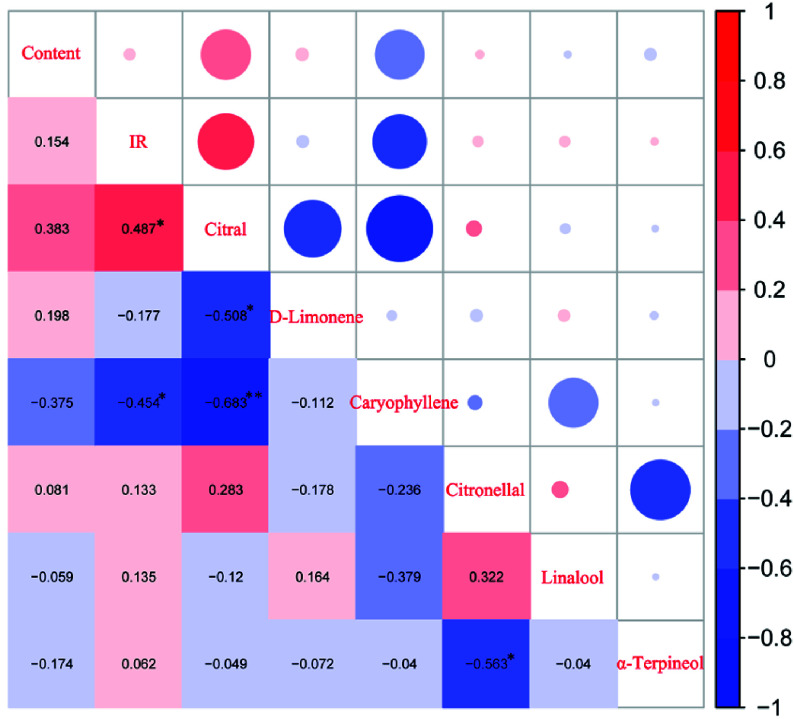
Correlation analysis between the primary components and the inhibition rates of *L. cubeba* oils. The cells of the lower left triangle and the circles of the upper right triangle represent the same values. Red colors indicate positive correlation, while blue colors indicate negative correlation. The correlation factors are labeled on the diagonal. The symbol '*' indicates a significant correlation (significance level of 5%), and '**' indicates a highly significant correlation (significance level of 1%).

In order to explore the relationship between antifungal activities and chemical compositions in *L. cubeba* oils, a correlation matrix was generated in R (Fig. 4). The six components utilized for the correlation analysis included citral (geranial and neral), D-limonene, caryophyllene, citronellal, linalool, and α-terpineol. The correlation analysis indicated that the main component, citral, was highly correlated with antifungal activities of *L. cubeba* oils, while caryophyllene was negatively correlated with antifungal activity. D-limonene and caryophyllene were significantly negatively correlated with citral, and α-terpineol was significantly negatively correlated with citronellal. In addition, there was a positive correlation between citral content and *L. cubeba* essential oil content, with a correlation coefficient of 0.383 (Fig. 4).

### Antibacterial activity assessments of *L. cubeba* families

**Figure 5:**
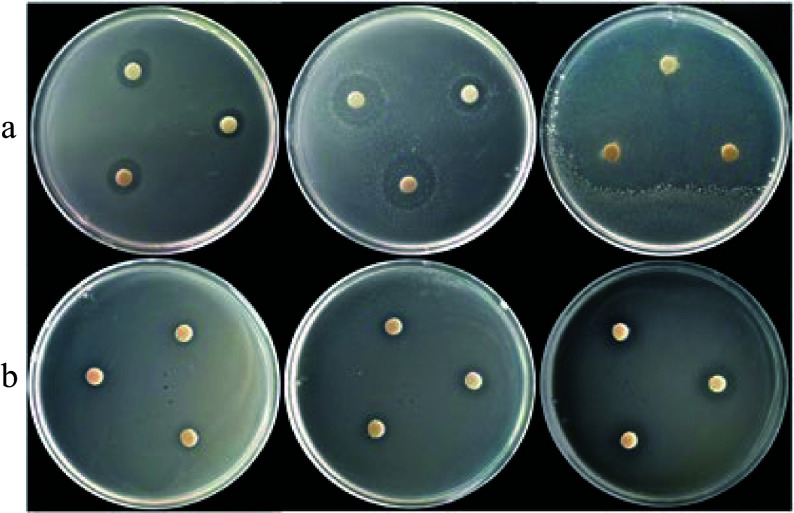
The antibacterial activity of *L. cubeba* essential oil from the G3 family against *E. coli* and *L. monocytogenes*. (a) Antibacterial effects of essential oil from the G3 family on *E. coli*. (b) Antibacterial effects of essential oil from the G3 family on *L. monocytogenes*. Essential oil concentrations from left to right are 25, 50, and 100 μL/mL. Petri dishes with a diameter of 90 mm were used.

**Figure 6:**
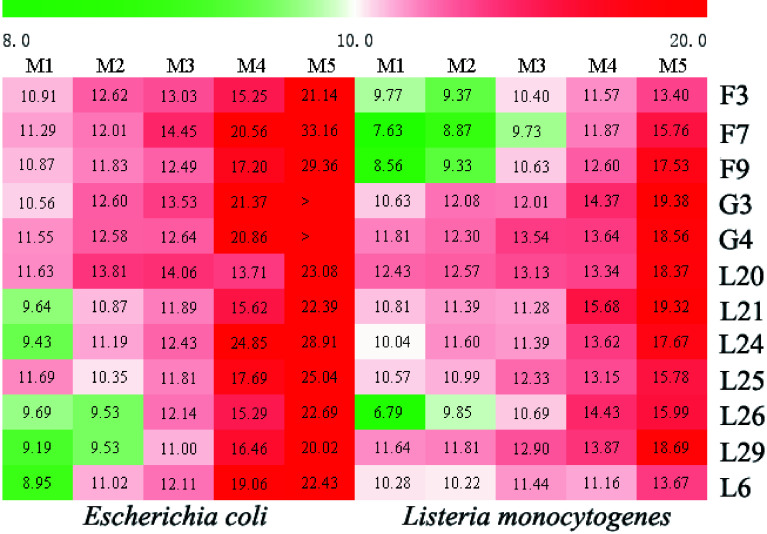
The inhibition rate of *L. cubeba* essential oil from various families with gradient concentration against bacteria. The inhibition rates of *L. cubeba* essential oils were analyzed according to the size of the inhibition zone diameter, then utilized to construct a heat map using R (www.r-project.org). The red background represents strong inhibitory effects, the white background represents moderately inhibitory effects, and the green background indicates no inhibitory effect. M1 to M5 represent the different concentrations (6.25, 12.5, 25, 50, and 100 μL/mL) of essential oil from each family. The inhibition zone indicates the diameter of the filter paper disk (6 mm).

Based on the antifungal activity of the essential oils, 12 families were assessed for antibacterial activities against *E. coli* and *L. monocytogenes*. The inhibition zone diameter method was used for antibacterial activity assessments. Antibacterial effects of the G3 family essential oil were found for both *E. coli* and *L. monocytogenes*, with a positive correlation seen between concentration and antibacterial activity (Fig. 5). In addition, *L. cubeba* oils had better antibacterial activity on *E. coli* compared to *L. monocytogenes*, with larger inhibition zones seen at all concentrations (Fig. 5). In order to further investigate the antibacterial effect of *L. cubeba* essential oil, antibacterial assessments in 12 *L. cubeba* families were carried out (Fig. 6). The inhibition effects were then grouped based on the size of their inhibition zone, including strong (20 mm), moderate (12−20 mm) and non-inhibitory (< 12 mm)^[13]^. When an essential oil concentration of 100 µL/mL was used, *E. coli* was extremely sensitive to all *L. cubeba* essential oils, while no obvious extreme inhibitory effects were observed for any kind of *L. cubeba* essential oil against *L. monocytogenes*. Furthermore, when the essential oil concentration was 50 µL/mL, the *L. cubeba* oils extracted from F7, G3, G4, and L24 had strong inhibitory effects on *E. coli*. Most essential oils, except F3 and L6, were moderately inhibitory for *L. monocytogenes* and showed a high sensitivity at a concentration of 100 µL/mL. Only L21 showed strong inhibitory effects against *L. monocytogenes* at a concentration of 50 µL/mL.

## DISCUSSION

### Citral is significantly positively correlated with antifungal activity

The essential oil of each family contained more than 30 components, most of which represented less than 5% of the total volume. Analysis of the correlation between the six primary oil components and antifungal activity of oils revealed that citral was significantly positively correlated with antifungal activity, while caryophyllene was significantly negatively correlated with antifungal activity (Fig. 5). The monoterpenes citral and citronellal have previously been shown to inhibit fungal growth^[14]^, which is in agreement with our results. In this study, caryophyllene content was negatively correlated with inhibition rate, despite the fact that Kim et al.^[15]^ found that rose bengal-sensitized photooxidation of *β*-caryophyllene could inhibit the growth of *Streptococcus aureus* and *Vibrio parahaemolyticus*. These differing findings could result from the fact that caryophyllene was negatively correlated with citral in *L. cubeba* oils, possibly due to direct or indirect competition for the synthesis of these two compounds. In addition, geraniol has been reported to have antifungal and fungicidal activity against *Penecillium digitatum*, *Penicillium italicum* and *Geotri-chum candidum*^[16]^, and nerol was shown to inhibit the growth of fungi^[17]^. With the exception of L30, L18, F5, F11, F24, F20, and F8, the essential oils of all families were composed of more than 80% citral, with some families reaching over 90% (Supplemental Table S1, S2). Citronellal represented more than 3% of the oil content from F25, L18, F2 and F4, while nerol and geraniol contents of F9, F20, F7, F11, F12, F5, F8, and F2 were the highest of the 31 *L. cubeba* oils. According to a previous study by Kumarsaikia et al.^[18]^, *L. cubeba* essential oil (LC5) collected in Itanagar, Arunachal Pradesh had a significantly different composition compared to LC3 (Sibsagar, Assam) and LC4 (Dibrugarh, Assam). LC5 was also shown to have higher activity against Gram-positive bacteria and yeast strains compared with LC3 and LC4, and we found that the components of LC5 were most similar to our *L. cubeba* essential oils. Taken together, our results indicate that citral is the driving force behind the antifungal activity of *L. cubeba* oils, but other components also play minor roles.

### Families with antimicrobial activity

In the present study, the vast majority of the 31 *L. cubeba* oils inhibited the growth of *Fof*-1 after 8 to 10 days of treatment with a concentration of 1,000 µL/L. This result is in agreement with previous research which found that the mycelial growth of *Aspergillus ﬂavus* was inhibited at a concentration of 1,000 µL/L^[9]^. As shown in Table 3, a significant proportion of *L. cubeba* oils had inhibition rates of more than 80%. *L. cubeba* oils extracted from F7, G3, G4, and F9 families had significant antifungal activities, with inhibition rates of up to 90% demonstrated at a concentration of 250 ul/L (Table 4). The MIC of oils extracted from F7, G3, and F9 were lower than 350 µL/L (Table 3), which are lower values than those previously reported for *L. cubeba* oils against *A. flavus* (500 µL/L)^[9]^. *E. coli* was also found to be more sensitive to *L. cubeba* oils than *L. monocytogenes*, which was consistent with previous reports indicating the sensitivity of *E. coli* to such treatments^[18]^. These differences may be due to the fact that *E. coli* is Gram-negative, while *L. monocytogenes* is Gram-positive, which may be a general trend that requires further investigation due to opposite trends seen in other studies by Mayaud et al.^[19]^. All samples except for F3 and L6 were found to have similar or stronger antibacterial properties compared to LC5. In general, the antibacterial activities of families F7, G3, G4, and L24 were strongest at low concentrations.

## CONCLUSIONS

*L. cubeba* has the highest content of citral among known essential oil crops, and our results indicate that citral was strongly correlated with antifungal and antibacterial activity. In this study, the F7, G3, G4, and F9 families were found to have strong antifungal activity, while the F7, G3, G4, and L24 families had strong antibacterial activity. These varieties could therefore be utilized to breed *L. cubeba* lines with improved antifungal and antibacterial properties in the future. Taken together, this study provides new insights into the mechanisms behind the antifungal and antibacterial activities of *L. cubeba* essential oils and can be utilized to further improve *L. cubeba* in future breeding efforts.

## MATERIALS AND METHODS

### Plant material

*L. cubeba* oils were separately extracted from 31 families of *L. cubeba* grown in an 8-year-old test forest in Liping county, Guizhou Province (Table 5). The forest was located at 26.133 degrees north and 109.233 degrees east, in a subtropical monsoon climate. The average annual rainfall of the test forest was 1,241 mm, with an annual average temperature of 16.4 °C and 298 frost-free days. All the samples were collected from different provenances in 2010 and then planted in Liping county, Guizhou Province in 2010. The fresh fruits of *L. cubeba* were collected from August to September in 2017 and were immediately preserved at 4 °C.

**Table 5:** The oil contents of 31 families from five provenances in a field experimental trial in Liping county, Guizhou Province.

Families	Essential oil content %	Provenance	Families	Oil yield %	Provenance
G3	4.48%	Dushan, Guizhou province	F9	3.09%	Jianyang, Fujian province
G4	6.56%	Dushan, Guizhou province	L6	12.05%	Bijie, Guizhou province
F21	2.39%	Anhui province	L7	6.44%	Bijie, Guizhou province
F1	5.00%	Fenyi, Jiangxi province	L9	5.41%	Bijie, Guizhou province
F2	4.30%	Fenyi, Jiangxi province	L30	2.35%	Fuyang, Zhejiang province
F3	3.22%	Fenyi, Jiangxi province	L24	5.57%	Jianou, Fujian province
F4	2.44%	Fenyi, Jiangxi province	L25	5.46%	Jianou, Fujian province
F12	2.98%	Fuyang, Zhejiang province	L26	4.80%	Jianou, Fujian province
F24	4.00%	Guangxi Zhuang Autonomous region	L27	1.96%	Jianou, Fujian province
F20	3.18%	Huangshan, Anhui province	L28	4.45%	Jianou, Fujian province
F25	2.44%	Jiangle, Fujian province	L29	5.09%	Jianou, Fujian province
F11	1.85%	Jianyang, Fujian province	L18	1.88%	Yuexi, Anhui province
F5	2.76%	Jianyang, Fujian province	L19	3.94%	Yuexi, Anhui province
F6	3.76%	Jianyang, Fujian province	L20	3.86%	Yuexi, Anhui province
F7	2.46%	Jianyang, Fujian province	L21	3.83%	Yuexi, Anhui province
F8	1.82%	Jianyang, Fujian province			

### Fungal and bacterial strains

*Fusarium oxysporum* f. sp. *fordii* 1 (Fof-1) was isolated from the root interior and rhizospheric soil of tung trees (*V. fordii*) with *Fusarium* wilt disease^[20]^, and further purification and culture were conducted using Potato Dextrose Agar (PDA) medium. *Escherichia coli* and *Listeria monocytogenes* were obtained from the lab of Doctor Da-Feng Song, at Zhejiang Sci-Tech University.

### Essential oil extraction from 31 families

The content of oil extracted from the fresh fruit of 31 families of *L. cubeba* was tested for three consecutive years, from 2014 to 2017. For each family, the fresh fruit samples of *L. cubeba* were boiled by steam distillation for more than 5 h^[8]^ and a Clevenger-type apparatus was used for the *L. cubeba* oil extraction and isolation. The essential oil in the supernatant was dried over anhydrous sodium sulfate (Na_2_SO_4_) and preserved at 4 °C. Samples from each family were treated separately. The essential oil content of *L. cubeba* was taken as the ratio of the weight of net essential oil to the weight of the fresh fruit.

### GC-MS analysis of *L. cubeba* oils

GC-MS was used for examining the chemical composition and content of *L. cubeba* oils. Before the GC-MS analysis, 50 ul of essential oil was dissolved in 5 mL of ethanol and then dehydrated by anhydrous sodium sulfate. The remaining liquid was then measured on a GC-MS instrument. A DB-5MS type chromatographic column which was 60 m long, with a 0.25 mm inner diameter and 0.25 µm thickness was utilized for the GC-MS experiments. An initial temperature of 50 °C was used for 2 min, followed by increases of 3 °C per min up to 80 °C for 2 min. The temperature was then increased by 5 °C per min up to 180 °C, 10 °C per min up to 230 °C and a final increase of 20 °C per min up to 250 °C for 3 min. A diversion ratio of 1:10 was used. High purity helium (99.999%) was used as the carrier gas at a constant flow rate of 1.5 mL/min. A total of 1 ul of sample was utilized for testing. The interface temperature of the GC-MS was 250 °C and the temperature of the ion source was 230 °C. The electron energy was 70 eV with a scanning mass range of 50 to 500 m/z. The relative retention time and the mass spectra were utilized for component identification of *L. cubeba* oil. All components were matched against the NIST08 standard spectrum library and relevant literature reports using their relative retention times. The relative content of each peak area was calculated by the peak area normalization method.

### Antifungal and antibacterial activity assessments of *L. cubeba* oil

#### Antifungal activity of *L. cubeba* oil on Fof-1

Pure *L. cubeba* oil which was extracted by water distillation was compounded to mix essential oil by dimethylsulfoxide (DMSO). Each type of *L. cubeba* oil was examined with five concentration gradients, including 6.25 µL/mL, 12.5 µL/mL, 25 µL/mL, 50 µL/mL and 100 µL/mL. The polyoxin (1%) was diluted with water to five concentration gradients, respectively 12.5 µL/mL, 25 µL/mL, 50 µL/mL, 100 µL/mL, 200 µL/mL. Hygromycin was dissolved in DMSO with five concentration gradients, including 12.5 mg/mL, 25 mg/mL, 50 mg/mL, 100 mg/mL and 200 mg/mL. PDA solid medium (potato 200 g, glucose 20 g, agar 15 g, distilled water 1,000 mL) was sterilized by 121 °C high temperature steam for 20 min, then used for culturing *Fof*-1. After cooling to 55 °C, 60 mL of media was added to each glass bottle, followed by addition of 600 µL essential oil, polyoxin, or hygromycin. The resulting mixtures were then poured into 5 × 90-mm petri dishes under sterile conditions. A sterilized 9-mm perforator was then used to add *Fof*-1 mycelium, which was cultured in the solid PDA medium for 120 h and broken into multiple fungal cakes. Finally, the fungal cakes were grown in petri dishes at 28 °C. Using the intersection method, the colony diameter was measured at 48, 72, 96, 120, 144, and 168 h.

The inhibition rates of *L. cubeba* oils were calculated based on the daily measurements of *Fof*-1 colony size. Virulence was calculated based on a regression curve of the logarithm of concentration and microbial growth rate. The regression coefficients (R^2^) were found to range from 0.9423 to 0.9999. MIC was identified as the minimum drug concentration that inhibited the growth of pathogenic fungi in the culture medium. EC50 was considered as the molar concentration of an inhibitor that resulted in 50% of the maximum possible response for that inhibitor. Based on the toxicity equation, the calculated corresponding concentration of the 'x' value was regarded as the MIC value when the 'y' value was set as one. Similarly, EC50 value was identified as the corresponding concentration of the 'x' value when the 'y' value was set as 0.5.

#### Antibacterial activities of *L. cubeba* oils on *Escherichia coli* and *Listeria monocytogenes*

The antibacterial activities of the *L. cubeba* oils were evaluated by the agar disc diffusion method^[18]^. A total of 100 µL of *E. coli* suspension (1x10^7^ CFU/mL) was cultured overnight, mixed with 5 mL LB (Luria-Bertani) semisolid medium, and then spread on a petri dish of pre-prepared solid LB medium (tryptone 10 g, yeast extract 5 g, NaCl 10 g, agar 15 g, distilled water 1000 mL). The essential oil of each *L. cubeba* family was dissolved in DMSO at five concentration gradients, including 6.25, 12.5, 25, 50, and 100 µL/mL. Round filter papers with 5 mm diameters containing 20 µL of essential oils were placed on the surface of petri dishes. After incubating at 37 °C for 24 h, the inhibition zone diameter was measured by a vernier caliper. *L. monocytogenes* was cultured in Brain Heart Infusion Broth (BHI) medium for antibacterial activity assessments of *L. cubeba* oil.

### Statistical analysis

All the experimental data were obtained from at least three replicates. The diameter of colony growth was calculated as the colony diameter minus the diameter of initial inoculum. The mycelial growth inhibition rate was calculated as the ratio of the difference of control and treated colony diameter to the diameter of control colony growth. The toxicity equation, correlation coefficient (r), minimum inhibitory concentration (MIC) and semi-maximum effect concentration (EC50) were calculated using Microsoft Excel. The Data Processing System (DPS 17.10) software was used for significance and correlation analyses. The least significant difference (LSD)^[21]^ test was used for analysis of variance (ANOVA). The R-Project (www.r-project.org) was used for correlation matrix analysis and visualization via the 'corrplot' function in the corrplot package.

## SUPPLEMENTARY DATA

Supplementary data to this article can be found online.
