# Supplementary material for: Antimicrobial activity of essential oils extracted from Litsea cubeba
Source: For Res (Fayettev). 2022 Feb 25;2:2. doi: 10.48130/FR-2022-0002 (PMC11524314; doi:10.48130/FR-2022-0002)
Supplement: Supplementary file 1 — Supplementary data to this article can be found online. [file FR-2022-0002-S1.zip › 10.48130_FR-2022-0002-Suppl-TableS1.pdf]

Table S1. Chemical composition of *Litsea cubeba* essential oils. A total of 31 families were analyzed in this study and the first 16 families were listed in this table.

Chemical composition	L26	L28	L9	L21	L7	L18	L24	L29	L30	L27	L19	L20	L25	L6	G3	G4
**Monoterpene hydrocarbons**	**2.87**	**2.74**	**4.12**	**4.14**	**6.05**	**9.15**	**1.65**	**10.55**	**9.00**	**2.22**	**4.49**	**4.04**	**8.41**	**0.19**	**7.96**	**11.92**
D-Limonene	1.99	2.14	3.45	3.30	4.67	7.81	1.09	9.40	7.65	1.36	3.76	3.31	7.13	0.19	7.34	9.64
Terpinolene	0.24	-	-	0.06	0.09	-	-	-	-	0.05	-	0.10	0.07	-	-	-
β-Myrcene	0.18	0.29	0.28	0.27	0.49	0.60	0.22	0.49	0.53	0.39	0.32	0.28	0.55	-	0.36	1.11
α-Pinene	0.16	0.04	0.10	0.03	0.07	0.22	0.13	0.08	0.26	0.03	0.03	0.03	0.09	-	0.07	0.41
β-Pinene	0.08	0.06	0.06	0.07	0.11	0.24	0.05	0.11	0.24	0.22	0.04	0.05	0.13	-	0.15	0.42
Carene	0.07	-	-	0.12	-	-	-	-	-	-	-	0.05	-	-	-	-
4,7-Methano-1H-indene, octahydro-	0.06	0.15	0.07	0.09	0.14	0.13	0.07	0.12	0.01	0.09	0.14	0.10	0.12	-	-	-
β-Phellandrene	0.02	0.01	0.02	0.06	0.23	0.06	0.02	0.25	0.16	0.01	0.07	0.03	0.19	-	-	-
Cyclohexene, 1-methyl-3-(1-methylethenyl)-,(.+/-.)-	0.01	0.02	-	-	0.17	0.01	0.02	-	-	0.04	0.05	-	-	-	-	-
Fenchene	0.01	-	-	-	-	0.02	-	0.03	0.06	-	-	0.01	-	-	-	-
Camphene	0.01	0.01	0.01	0.01	0.01	0.03	0.05	0.04	0.06	0.00	0.00	0.01	0.01	-	-	0.09
(+)-4-Carene	0.01	0.01	0.02	0.04	0.03	0.04	0.01	0.03	0.03	0.02	0.02	0.03	0.05	-	-	-
α-Phellandrene	0.01	0.02	0.12	0.10	0.04	-	-	-	-	-	0.05	0.04	0.07	-	-	-
Bicyclo[3.1.0]hex-2-ene, 4-methyl-1-(1-methylethyl)-	-	-	-	-	-	-	-	-	-	-	-	-	-	-	-	0.15
2H-Inden-2-one, 1,4,5,6,7,7a-hexahydro-7a-methyl-, (S)-	-	-	-	-	-	-	-	-	-	-	-	-	-	-	-	0.04
1,3-Cyclohexadiene, 1-methyl-4-(1-methylethyl)-	-	-	-	-	-	-	-	-	-	-	-	-	-	-	0.05	-
1,3,6-Heptatriene, 2,5,6-trimethyl-	-	-	-	-	-	-	-	-	-	-	-	-	-	-	-	0.06

(Continued)

Table S1. (Continued).

Chemical composition	L26	L28	L9	L21	L7	L18	L24	L29	L30	L27	L19	L20	L25	L6	G3	G4
**Oxygenated monoterpenes**	**91.10**	**95.18**	**93.96**	**92.64**	**90.88**	**87.68**	**95.07**	**86.33**	**84.86**	**94.59**	**92.74**	**89.97**	**86.91**	**95.64**	**88.29**	**85.42**
Geranial	46.73	48.95	48.66	46.83	46.08	42.54	46.30	43.41	42.55	46.77	46.87	45.31	43.47	50.00	46.44	45.96
Neral	39.20	41.64	41.05	40.61	39.99	37.09	44.00	37.22	38.08	43.46	40.73	40.18	38.47	39.97	37.36	35.36
Limonene epoxide	1.54	1.96	1.48	2.15	1.85	1.64	1.76	1.64	1.57	1.66	1.87	1.87	1.95	-	0.14	-
Citronellal	1.06	0.80	1.13	0.72	0.75	4.12	0.45	0.68	0.82	0.79	0.84	0.67	0.69	1.04	0.54	0.56
Linalool	0.67	1.38	0.62	0.85	1.29	1.01	1.29	0.95	1.28	0.94	1.25	0.68	0.74	1.36	1.35	1.25
2-Cyclohexen-1-ol,1-methyl-4-(1-methylethenyl)-, trans-	0.46	-	-	-	0.09	0.12	0.23	0.12	-	-	0.11	-	-	-	-	-
Acetaldehyde, (3,3-dimethylcyclohexylid-ene)-, (Z)-	0.38	0.01	0.05	0.30	0.06	0.04	0.06	0.14	-	0.31	0.18	0.19	0.18	-	-	-
α-Terpineol	0.19	0.09	0.62	0.03	0.08	0.08	0.05	1.36	0.24	0.09	0.14	0.25	0.26	0.40	0.62	0.31
Nerol	0.17	0.01	0.06	0.30	0.15	0.29	0.23	0.24	-	-	0.13	0.04	0.45	-	0.26	-
2,6-Octadienal, 3,7-dimethyl-, (E)-	0.15	0.07	-	-	-	0.05	0.05	-	-	-	-	-	-	-	-	-
(S)-cis-Verbenol	0.13	0.02	0.04	-	0.03	0.05	0.02	0.08	0.01	0.08	0.05	0.09	0.03	0.62	0.63	0.05
Geraniol	0.11	0.00	-	0.15	0.07	0.37	0.23	0.14	-	-	0.21	-	-	0.29	0.34	0.43
2-Isopropenyl-5-methylhex-4-enal	0.10	0.11	0.19	0.33	0.18	-	0.38	0.12	0.28	0.32	0.11	0.26	0.48	-	-	-
Isoborneol	0.07	-	-	0.34	0.01	0.00	0.03	-	-	-	-	0.06	-	-	-	-
2,6-Octadien-1-ol, 3,7-dimethyl-, (Z)-(Z)-	0.05	-	-	0.00	0.01	0.01	0.01	-	0.01	0.03	0.02	0.00	0.01	-	-	-
p-menth-8-en-3-one	0.04	-	0.03	-	-	0.02	-	0.17	-	-	0.06	-	-	-	-	-
Bicyclo[3.1.1]hept-3-en-2-ol, 4,6,6-trimethyl-	0.04	-	-	-	0.04	0.02	-	0.04	-	-	0.03	0.16	0.03	-	-	-
cis-Carveol	0.00	0.01	0.00	0.00	-	0.00	-	-	-	-	-	-	-	-	-	-
(+)-cis-sabinol	-	0.12	0.01	0.03	0.19	0.06	0.01	-	0.01	0.14	0.12	0.20	0.14	-	-	-
(+)-Citronellal	-	0.00	0.03	0.01	-	0.15	-	0.02	0.00	-	0.03	0.00	0.00	-	-	-

(Continued)

Table S1. (Continued).

Chemical composition	L26	L28	L9	L21	L7	L18	L24	L29	L30	L27	L19	L20	L25	L6	G3	G4
Bicyclo[2.2.1]heptan-2-one, 1,7,7trimethyl-, (1S)-	-	-	-	-	-	-	-	-	-	-	-	-	-	-	0.02	-
Terpineol, cis-.beta.-	-	-	-	-	-	-	-	-	-	-	-	-	-	-	-	0.04
Eucalyptol	-	-	-	-	-	-	-	-	-	-	-	-	-	0.03	0.48	0.95
Neric acid	-	-	-	-	-	-	-	-	-	-	-	-	-	0.29	-	-
Pulegone	-	-	-	-	-	-	-	-	-	-	-	-	-	1.19	-	0.40
3-Cyclohexen-1-one, 2-isopropyl-5-methyl-	-	-	-	-	-	-	-	-	-	-	-	-	-	0.24	-	-
2-Cyclohexen-1-one, 3-methyl-6-(1-methylethyl)-	-	-	-	-	-	-	-	-	-	-	-	-	-	-	0.06	0.04
Borneo camphor	-	-	-	-	-	-	-	-	-	-	-	-	-	0.04	0.06	0.06
6-Octen-1-ol, 3,7-dimethyl-, (R)-	-	-	-	-	-	-	-	-	-	-	-	-	-	0.10	-	-
Cyclohexanemethanol, 4-hydroxy-.alpha.,.alpha.,4-trimethyl-	-	-	-	-	-	-	-	-	-	-	-	-	-	0.10	-	-
**Sesquiterpenes**	**2.97**	**0.21**	**0.29**	**1.06**	**0.74**	**0.38**	**1.21**	**1.07**	**4.10**	**1.30**	**0.79**	**1.78**	**1.57**	**0.48**	**0.31**	**0.25**
Caryophyllene	1.55	0.10	0.13	0.55	0.40	0.06	0.47	0.31	0.79	0.61	0.35	1.03	0.70	0.48	0.27	0.25
Caryophyllene oxide	0.74	0.04	0.01	0.09	0.06	0.04	0.02	0.05	0.06	0.01	0.12	0.11	0.03	-	-	-
Farnesol	0.29	-	0.05	0.08	0.07	0.03	0.13	0.07	-	-	0.08	-	-	-	-	-
Nerolidol	0.17	0.02	0.06	0.17	0.13	0.12	0.35	0.13	0.22	-	0.10	0.11	0.23	-	-	-
β-selinene	0.13	-	-	0.00	-	-	-	-	-	0.00	-	0.01	-	-	-	-
Farnesol isomera	0.05	0.00	0.02	0.09	0.01	0.04	-	0.08	2.21	-	0.05	-	0.03	-	-	-
(E)-β-Farnesene	0.05	0.01	0.02	0.04	0.04	0.03	0.00	0.01	0.01	0.05	-	0.01	0.02	-	-	-

(Continued)

Table S1. (Continued).

Chemical composition	L26	L28	L9	L21	L7	L18	L24	L29	L30	L27	L19	L20	L25	L6	G3	G4
(-)-β-Elemene	-	0.04	0.00	0.04	0.03	0.06	0.24	0.43	0.81	0.63	0.09	0.51	0.56	-	-	-
1H-Cycloprop[e]azulen-7-ol, decahydro-1,1,7-trimethyl-4-methylene-, [1ar-(1a.alpha.,4a.alpha.,7.beta.,7a.beta.,7b.alpha.)]-	-	-	-	-	-	-	-	-	-	-	-	-	-	-	0.03	
**Diterpenes**	**0.05**	-	-	-	-	**0.43**	-	-	-	-	-	-	-	-	-	-
Geranylgeraniol	0.05	-	-	-	-	0.43	-	-	-	-	-	-	-	-	-	-
**Others**	**2.19**	**1.77**	**1.52**	**1.73**	**2.33**	**2.36**	**2.07**	**2.05**	**2.04**	**1.88**	**1.98**	**4.22**	**3.09**	**0.34**	**1.25**	**1.95**
2-Cyclohexen-1-one,4-(2-oxopropyl)-	0.82	1.09	0.94	1.07	1.07	1.17	0.99	0.98	0.96	0.97	1.05	1.06	1.10	-	-	-
3-Cyclopentene-1-acetaldehyde, 2-oxo-	0.42	-	-	0.08	0.19	0.17	-	0.11	0.24	0.25	0.11	0.04	0.17	-	-	-
Cyclohexanepropanal, 2,2-dimethyl-6-methylene-	0.34	0.02	0.05	0.16	0.23	0.04	0.44	0.21	-	-	0.08	-	0.24	-	-	-
5-Hepten-2-one	0.24	0.40	0.36	0.23	0.51	0.45	0.27	0.52	0.51	0.39	0.46	0.38	0.42	0.15	0.99	1.58
Cyclopropane, 1,1-dimethyl-2-(2-propenyl)-	0.16	0.19	0.14	0.17	0.19	0.15	0.15	0.16	0.14	0.14	0.18	0.71	0.15	-	-	-
(+)-Epi-bicyclosesquiphellandrene	0.06	0.04	0.00	0.00	0.02	0.03	0.02	0.01	-	0.04	0.01	-	0.01	-	-	-
Geranyl phenylacetate	0.05	0.00	-	-	-	0.02	-	-	-	-	-	1.90	0.82	-	-	-
2,6,10,14-Hexadecatetraen-1-ol, 3,7,11,15-tetramethyl-, acetate, (E,E,E)-	0.04	-	-	-	-	0.16	-	-	-	-	-	-	-	-	-	-
Farnesol, acetate	0.03	-	-	-	-	0.00	-	-	-	-	-	-	-	-	-	-
Bicyclo[5.1.0]octane, 8-(1-methylethylidene)-	0.01	0.01	0.02	0.00	0.01	0.01	0.01	0.01	0.01	0.00	0.01	0.01	0.01	-	-	-
Cyclohexane, 1,2-diethenyl-, cis-	0.01	0.01	0.02	-	0.04	0.03	-	-	-	-	0.04	-	0.01	-	-	-
Styrene	0.01	0.01	0.01	0.01	0.01	0.04	0.05	0.03	0.01	0.00	0.00	0.00	0.00	-	-	-
1H-Imidazole,1-ethyl-2-methyl	-	-	-	-	-	-	-	-	-	-	-	-	-	0.10	-	-
Benzenamine, 3-ethoxy-	-	-	-	-	0.06	0.08	0.14	0.02	0.12	0.09	0.04	0.06	0.15	-	-	-
5,5-Dimethyl-1,3-hexadiene	-	-	-	-	0.01	-	0.01	-	0.06	-	-	0.05	-	-	-	-
methylheptenol	-	-	-	-	-	-	-	-	-	-	-	-	-	-	0.04	-

(Continued)

Table S1. (Continued).

Chemical composition	L26	L28	L9	L21	L7	L18	L24	L29	L30	L27	L19	L20	L25	L6	G3	G4
Methyl salicylate	-	-	-	-	-	-	-	-	-	-	-	-	-	-	0.04	-
Naphthalene, decahydro-1,5-dimethyl-	-	-	-	-	-	-	-	-	-	-	-	-	-	0.04	0.03	-
4 - terpene alcohols	-	-	-	-	-	-	-	-	-	-	-	-	-	0.05	0.07	-
.gamma.-Elemene	-	-	-	-	-	-	-	-	-	-	-	-	-	-	-	0.04
Cyclopropanemethanol, 2-methyl-2-(4-methyl-3-pentenyl)-	-	-	-	-	-	-	-	-	-	-	-	-	-	-	-	0.33
7-Propylidene-bicyclo[4.1.0]heptane	-	-	-	-	-	-	-	-	-	-	-	-	-	-	0.05	-
trans-p-Mentha-2,8-dienol	-	-	-	-	-	-	-	-	-	-	-	-	-	-	0.03	-
