# Supplementary material for: Antimicrobial activity of essential oils extracted from Litsea cubeba
Source: For Res (Fayettev). 2022 Feb 25;2:2. doi: 10.48130/FR-2022-0002 (PMC11524314; doi:10.48130/FR-2022-0002)
Supplement: Supplementary file 1 — Supplementary data to this article can be found online. [file FR-2022-0002-S1.zip › 10.48130_FR-2022-0002-Suppl-TableS2.pdf]

Table S2. Chemical composition of *Litsea cubeba* essential oils. A total of 31 families were analyzed in this study and the last 15 families were listed in this table.

Chemical composition	F1	F2	F3	F4	F5	F6	F7	F8	F9	F11	F12	F20	F21	F24	F25
**Monoterpene hydrocarbons**	**3.48**	**0.73**	**3.76**	**0.57**	**2.38**	**3.96**	**0.22**	**10.16**	**0.03**	**1.12**	**0.09**	**0.36**	**0.37**	**10.68**	**1.17**
D-Limonene	3.02	0.60	3.29	0.34	1.77	3.16	0.19	7.27	0.03	0.65	0.08	0.24	0.29	8.98	0.88
α-Pinene	0.02	-	0.02	-	0.02	0.06	-	0.48	-	0.06	-	-	0.01	0.37	-
β-Pinene	0.06	-	0.05	-	0.07	0.09	0.01	0.85	-	0.10	-	0.01	0.01	0.42	0.01
β-Phellandrene	0.17	0.06	0.19	-	0.15	0.36	0.02	0.81	-	0.16	-	-	-	0.63	0.07
Camphene	0.01	-	-	-	-	0.03	-	0.08	-	0.01	-	-	-	0.09	-
(+)-4-Carene	0.02	-	0.01	-	0.03	0.04	-	0.09	-	-	-	-	-	0.03	0.02
Bicyclo[4.1.0]hept-2-ene, 3,7,7-trimethyl-	-	-	-	-	0.01	-	-	0.10						0.01	-
Carvone oxide, trans-	-	-	0.02	0.03	0.04	0.04	-	-	-	0.02	-	-	-	-	0.03
Benzene, 1-methyl-2-(1-methylethyl)-	-	-	0.01	-	0.02	-	-	0.08	-	0.04	-	-	-	0.01	-
.gamma.-Terpinene	-	-	0.01	-	0.06	-	-	0.28	-	0.03	0.01	0.01	-	0.02	0.01
Bicyclo[2.2.1]heptane, 7,7-dimethyl-2-methylene-	0.13	0.07	0.16	0.20	0.21	0.18	-	0.12	-	-	-	0.10	0.06	0.08	0.11

(Continued)

Table S2. (Continued).

Chemical composition	F1	F2	F3	F4	F5	F6	F7	F8	F9	F11	F12	F20	F21	F24	F25
1,6-Heptadiene, 2,5,5-trimethyl-	0.05	-	-	-	-	-	-	-	-	0.05	-	-	-	0.04	0.04
Geranial	47.85	48.03	46.76	45.91	43.06	45.46	47.13	36.74	47.55	40.50	46.47	37.59	45.72	38.90	42.98
Neral	38.72	38.31	36.84	36.62	35.33	36.55	38.04	31.89	38.36	31.81	37.35	31.91	35.69	32.03	37.23
Limonene epoxide	-	0.03	0.12	0.16	0.24	0.20	0.03	0.06	0.03	0.06	0.04	0.07	-	0.04	0.11
Citronellal	0.81	3.95	1.71	3.19	1.05	1.21	0.64	0.73	0.68	0.49	0.57	0.63	0.65	0.92	4.58
Linalool	1.38	1.47	1.30	1.35	1.66	1.65	1.46	1.88	1.49	1.20	1.50	1.48	0.72	1.26	1.92
α-Terpineol	1.32	0.37	1.14	1.27	1.45	0.35	1.37	1.21	2.26	1.68	2.01	1.19	0.58	1.35	0.47
Nerol	0.35	0.84	0.45	0.43	0.97	0.50	1.61	0.91	1.67	1.08	1.06	1.63	0.28	0.44	0.49
(S)-cis-Verbenol	-	-	0.03	0.05	0.09	0.06	0.03	0.05	0.05	0.04	0.05	0.04	-	0.02	0.03
Geraniol	0.73	1.41	0.73	0.49	1.96	0.79	3.18	1.05	1.81	1.18	2.71	3.24	0.00	0.82	0.71
Bicyclo[3.1.1]hept-3-en-2-ol, 4,6,6-trimethyl-	0.95	1.00	0.93	0.67	0.82	0.95	0.73	1.15	0.89	0.31	0.74	0.64	0.52	0.69	0.79
cis-Carveol	0.03	-	0.06	0.10	0.12	0.13	0.04	0.03	0.03	0.06	0.03	0.05	0.02	0.02	0.05
Bicyclo[2.2.1]heptan-2-one, 1,7,7-trimethyl-, (1S)-	-	-	-	0.04	-	-	0.05	-	-	0.03	-	-	-	-	0.05
5,7-Octadien-2-ol,2,6-dimethyl-	0.08	0.07	-	0.14	0.12	0.18	-	-	0.11	-	0.12	-	0.28	0.19	0.11
2-Cyclohexen-1-ol, 2-methyl-5-(1-methylethenyl)-, trans-	-	-	-	0.07	-	0.13	-	-	-	-	-	-	0.07	0.09	-
Terpineol, cis-.beta.-	-	-	-	-	0.04	0.02	0.02	0.02	0.07	-	0.05	0.04	-	-	0.01
Cyclohexanone, 5-methyl-2-(1-methylethenyl)-, trans-	1.32	1.40	1.34	1.04	1.19	1.50	1.09	1.90	1.24	0.66	1.11	0.95	0.90	1.08	1.07
Eucalyptol	1.11	0.19	0.50	0.13	0.56	0.01	0.20	2.44	0.06	0.58	0.14	0.21	-	1.43	0.24
Oxiranecarboxaldehyde,3-methyl-3-(4-methyl-3-pentenyl)-	0.09	-	0.19	0.34	0.28	0.32	0.14	-	-	0.12	0.11	0.13	0.06	0.07	0.18
Piperitone	0.12	0.11	0.15	0.17	0.14	0.16	0.14	0.13	0.11	0.25	0.12	0.15	0.12	0.10	0.12

(Continued)

Table S2. (Continued).

Chemical composition	F1	F2	F3	F4	F5	F6	F7	F8	F9	F11	F12	F20	F21	F24	F25
3-Cyclohexen-1-ol, 4-methyl-1-(1-methylethyl)-	0.13	0.07	0.15	0.14	1.00	0.08	0.71	1.09	0.77	0.64	1.01	0.72	0.03	0.10	0.27
Borneol	0.14	0.10	0.11	0.07	0.18	0.10	0.12	0.06	0.14	0.13	0.12	0.12	0.13	0.07	
3-Oxatricyclo[4.1.1.0(2,4)]octane, 2,7,7-trimethyl-	-	0.03	0.08	0.15	0.12	0.14	0.03	0.01	0.02	0.03	0.02	0.04	0.01	0.02	0.06
Neric acid	-	-	0.06	0.04	0.02	0.06	-	0.02	0.02	0.10	-	-	0.03	0.04	-
Geranic acid	-	-	0.06	0.06	0.04	0.09	-	0.04	0.03	0.18	0.04	0.10	0.08	0.05	-
Carveol	-	0.06	0.03	0.05	0.07	0.08	0.04	0.10	0.06	0.07	0.08	0.08	0.05	0.05	0.05
**Sesquiterpenes**	**0.65**	**0.72**	**1.02**	**2.39**	**2.72**	**2.29**	**0.95**	**1.52**	**0.87**	**5.72**	**1.47**	**6.13**	**4.73**	**3.21**	**2.58**
Caryophyllene	0.53	0.66	1.00	1.95	2.16	1.50	0.90	1.51	0.85	5.47	1.43	6.03	4.53	3.15	2.51
Caryophyllene oxide	0.12	0.06	0.02	0.38	0.54	0.75	0.05	0.01	0.02	0.23	0.04	0.10	0.20	0.06	0.07
2Z,6E-Farnesol	-		-	0.06	0.02	0.04	-	-	-	0.02	-	-	-	-	-
**Others**	**0.74**	**1.12**	**2.48**	**4.35**	**4.41**	**3.05**	**2.04**	**6.80**	**1.65**	**11.96**	**3.00**	**12.45**	**8.83**	**6.34**	**4.72**
5-Hepten-2-one	0.03	-	-	-	-	0.10	0.04	0.46	0.02	-	0.05	0.11	-	0.09	0.04
Butanoic acid, 3,7-dimethyl-6-octenyl - ester	-	-	0.01	0.02	0.02	0.02	-	-	-	-	-	-	-	-	0.03
2,6-Octadiene, 1,1-diethoxy-3,7-dimethyl-	0.44	0.84	2.16	3.99	3.94	2.52	1.83	2.70	1.46	11.55	2.68	12.07	8.68	6.00	4.34
2-Octene, 2-methyl-6-methylene-	0.14	0.23	0.17	0.17	0.17	0.22	0.12	0.23	0.16	0.10	0.13	0.12	0.10	0.12	0.14
Tricyclo[2.2.1.0(2,6)]heptane-3methanol, 2,3-dimethyl-	0.11	0.05	0.12	0.17	0.16	0.19	0.02	0.05	-	0.07	-	0.05	0.05	0.02	0.15
Cyclohexene, 4-methylene-1-(1-methylethyl)-	0.02	-	0.02	-	0.12	-	0.03	3.36	0.01	0.23	0.01	0.04	-	0.11	-
Cyclohexane, (1-methylethyl)-	-	-	-	-	-	-	-	-	-	0.01	0.13	0.06	-	-	0.02
