# Supplementary material for: Antimicrobial activity of essential oils extracted from Litsea cubeba
Source: For Res (Fayettev). 2022 Feb 25;2:2. doi: 10.48130/FR-2022-0002 (PMC11524314; doi:10.48130/FR-2022-0002)
Supplement: Supplementary file 1 — Supplementary data to this article can be found online. [file FR-2022-0002-S1.zip › 10.48130_FR-2022-0002-Suppl-FigureS1.pdf]

Screening of *Litsea cubeba* essential oil in families: chemical composition and antimicrobial activity

Supplementary Materials

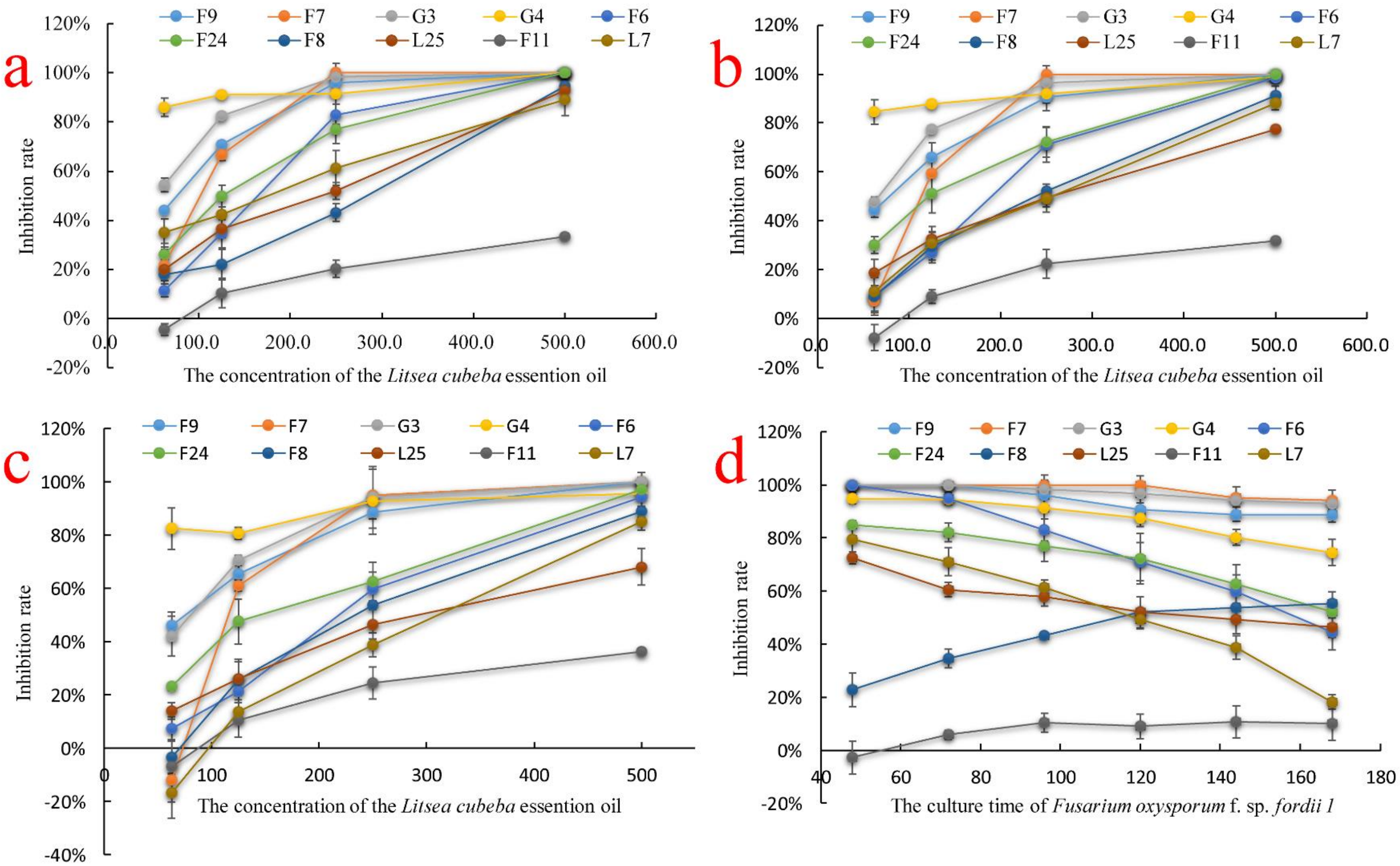

Fig. S1. The trends in inhibition rates under different concentrations or culture times against *Fof*-1. The abscissa axis represents concentration of essential oil in graph “a”, “b” and “c” and the vertical axis represents inhibition rate. In graph “d”, the abscissa axis represents culture time of *Fof*-1and the vertical axis represents

inhibition rate. Lines of different colors represent the *L. cubeba* oils in F9, F7, G3, G4, F6, F24, F8, L25, F11 and L7 families. The graph "a", "b" and "c" showed that the antifungal curve varied with *L. cubeba* oil concentrations (μL/L) at 96, 120 and 144 h, respectively. The graph "d" showed the changes of inhibition rate followed the culture time at 250 μL/L essential oil concentration.
